# Service user and stakeholder engagement in maternal and newborn health research in low- and middle-income countries: A systematic review protocol

**DOI:** 10.1371/journal.pone.0286145

**Published:** 2023-05-23

**Authors:** Devendra Raj Singh, Rajeeb Kumar Sah, Bibha Simkhada, Zoe Darwin

**Affiliations:** 1 Department of Public Health, Central Institute of Science and Technology (CIST) College, Pokhara University, Kathmandu, Nepal; 2 School of Human and Health Sciences, University of Huddersfield, Huddersfield, United Kingdom; HERD International, NEPAL

## Abstract

**Background:**

Service user and stakeholder engagement have been widely considered as key aspects in translating knowledge into realistic policies and practices. However, there is a paucity of accumulative evidence about service user and stakeholder engagements in maternal and newborn health (MNH) research in low- and middle-income countries (LMICs). Therefore, we aim to systematically review the existing literature that includes service user and stakeholder engagement in maternal and newborn health research in low- and middle–income countries.

**Materials and methods:**

The design of this protocol is guided by the Preferred Reporting Items for Systematic Reviews and Meta-Analysis (PRISMA-P) checklist. We will systematically run the search in PubMed/MEDLINE, PsycINFO, Scopus, Science Direct, and CINAHL to obtain relevant peer-reviewed literature published between January 1990 and March 2023. The list of extracted references will be screened by applying the study inclusion criteria, and eligible studies will be processed for further evaluation before being included in the review. The quality of the selected study will be assessed using the critical appraisal skills program (CASP) checklists and the Mixed Method Appraisal Tool (MMAT) checklist. A narrative synthesis will be used to synthesised results from all the included studies.

**Discussion and conclusion:**

To the best of our knowledge, this systematic review will be the first synthesised evidence on service user and stakeholder engagement in maternal and newborn health research in low- and middle-income countries. The study highlights the importance of service user and stakeholder roles in designing, implementing, and evaluating maternal and newborn health interventions in resource-poor settings. The evidence from this review is expected to be useful for national and international researchers/stakeholders for practising meaningful and effective ways of engaging users and stakeholders in maternal and newborn health research and related activities. The PROSPERO registration number is CRD42022314613.

## Background

Service users and other key stakeholders’ engagement in maternal and newborn health (MNH) services research have been recognised as crucial for developing, implementing, and evaluating evidence-based pragmatic solutions in different contexts [1–4]. The World Health Organization (WHO) explicitly states that the stakeholder’s engagement in MNH is essential to bringing wider inter-sectorial collaboration, joint learning and synergies, the right to participate and empower women, developing acceptable and respectful people-centred MNH services, and enhancing ownership of MNH improvement initiatives [5]. Stakeholders in the MNH research area include patients/service users, health professionals, community members, policy-makers, civil society, opinion leaders, and the private sectors who have a direct or indirect interest in the research outcome [5]. In this review, the service users/patients refer to pregnant women and postnatal/lactating mothers. Likewise, other key stakeholders will be grouped into family, community/local, state, national and international levels those who are concerned with maternal and newborn health. The concept of engaging service users and other key stakeholders in health research is embedded within the principle that their active participation can enhance the quality of research and help to minimise the gaps between research production and research application to improve interventions, services and health policy [6–8]. However, researchers are faced with challenges in bringing together the main groups of health system stakeholders and key actors beyond the health sectors [9]. Therefore, the engagement of service users and stakeholders in health research can be achieved in different forms and at different levels depending upon the nature of the study, its objectives, methods, and the context in which the research is undertaken [10, 11]. The term “stakeholder engagement” is defined as *“an iterative process of actively soliciting the knowledge*, *experience*, *judgment*, *and values of individuals selected to represent a broad range of direct interest in a particular issue*, *for the dual purposes of creating a shared understanding; making relevant*, *transparent and effective decisions”* [12]. Stakeholder engagement is also widely considered as a practice of democratic procedure that helps to provide legitimacy to the collaborative and partnership working to improve services [13, 14]. Also, researchers and practitioners believe that stakeholder engagement in health research is necessary to ensure the relevance of research projects and the usability of the produced evidence in the policy and practice [2, 10, 14].

The term “user and stakeholder engagement” in health research often refers to “patient and public involvement” (PPI), as defined by INVOLVE in the United Kingdom (UK) [15]. However, in this review, the concept of PPI is perceived as one aspect of stakeholder engagement within the broader concept [11, 16]. The International Association for Public Participation highlighted that stakeholder engagement could happen at five different levels: inform, consult, involve, collaborate and empower [17]. Thus, the engagement can refer to a range of activities such as the involvement of service users and stakeholders in identifying the local health needs, prioritising the research, inputs in designing and conducting the research, utilising the evidence for co-design of the policy, or intervention, implementation and evaluation [12, 14]. The collaborative approach of working with service users and stakeholders has been found to have a greater impact on achieving the expected outcomes [8]. However, the researcher’s understanding of the inner (within the organisation) and outer contexts (outside the organisation) of the setting where stakeholder engagements take place is essential for bringing multiple stakeholders to a common platform [18].

Despite considering multi-stakeholder roles as essential factors in the success of health interventions, the genuine partnership or meaningful engagement of stakeholders in MNH research is often lacking, particularly in the context of the low- and middle-income countries (LMICs) [19]. The partnership approach in research (participatory research), including co-design, co-production, and co-creation, advocates for creating a conducive environment for practicing equal levels of relationship between service users and stakeholders [20, 21]. However, understanding the extent to which power differences are shaped in the process of users’ and stakeholders’ engagement requires considerable aggregation of evidence from different settings. In this context, there are several important aspects of power dynamics that need to be examined, particularly in resource-poor countries. For example, how does the stakeholder’s engagement process lead to the empowerment of users or act as a facilitator or barrier for improving the health service uptake and delivery in different socio-cultural and political contexts. Thus, stakeholder engagements in research require a broader understanding of socio-cultural and participants’ power dynamics, especially when involving women with other stakeholders in the health research process in the patriarchal society of the LMICs [10]. Furthermore, there is an urgent need to identify the levels of or various ways in which service users and other key stakeholders have been engaged in MNH research in the LMICs [14, 22]. Hence, it is essential to review the evidence on how researchers in MNH research have employed the concept of stakeholder engagement in a resource-poor context, considering factors such as study setting, socio-cultural and political environment, stakeholder motivation, lack of awareness of collaborative work, and gendered power imbalance pose various challenges [22]. This systematic review will therefore review different approaches to stakeholder engagement in maternal and newborn health research in the low-and middle-income countries context.

### Rationale for systematic review

Maternal and newborn health is still a major public health concern in LMICs [23]. The current pace of decline in maternal and newborn deaths in resource-poor countries poses a great challenge in achieving the Sustainable Development Goal (SDG) targets 3.1 and 3.2. [5, 24]. In order to meet the MNH targets of SDGs by 2030, innovative and tailored MNH interventions are needed to scale up extensively to reach the unreached groups’ [24]. In addition, the abatement in the availability of double standards of MNH care and widespread disparities in accessing quality care among women and newborns in LMICs is fundamental to accelerating the MNH progress [25, 26]. The shortage of health workforce, poor accessibility and quality of health services, complex socio-cultural environment, the double standard of care, and fragile health system remain key challenges in improving delivery and uptake of MNH services in the LMICs [23, 25, 27]. Considering the complex nature of MNH problems and scarcity of resources, diverse approaches have been attempted to involve or engage service users and stakeholders in defining the problem and design, implementation, and evaluation of the MNH interventions [3, 28–30]. There is also ample published literature using the PPI concept in the area of MNH research in the LMICs [29–31]. However, there is a paucity of synthesised evidence on how such an approach has been used in the area of MNH research, particularly in LMICs. The World Health Organization has also recommended integrating stakeholders and community engagement in improving the quality of care for maternal, newborn, and child health [5]. Nonetheless, in some cases, stakeholder engagements were just found to be tokenistic in the research project for meeting the requirements of funding agencies [32]. The stakeholder’s engagement in such a context is mostly bureaucratic or expert deliberation where stakeholders have a limited practice of their autonomy [33]. This kind of disparagement has created an urgency to generate an accumulative piece of evidence to elucidate the definitive scenario of practicing stakeholders’ engagement in maternal and newborn health research in LMICs. This evidence will further be supportive of directives toward developing effective MNH initiatives and move on the way to meeting the SDGs targets 3.1 & 3.2 [5].

The evidence-based policy and practice for improving MNH services delivery and uptake, particularly in resource-poor countries, requires a comprehensive understanding of the approaches used and the perspectives of end-users and other stakeholders concerning their MNH needs [34–36]. Also, it is equally important to identify the gaps in the process of involving the service users and other stakeholders in the MNH research [10]. This review will provide updated evidence for engaging users and stakeholders while developing realistic MNH initiatives in the future, particularly in the context of LMICs. The review will also bring together the contextual evidence of service users’ and stakeholders’ engagement in maternal and newborn health that will be valuable for researchers, policymakers, planners, and funders to be prudent in engaging and involving service users and stakeholders in future MNH research. Based on our knowledge, this systematic review will be the first of its kind to generate synthesised evidence on service users’ and stakeholders’ engagement in MNH research in the context of LMICs. This review will fill the gap of knowledge regarding service users’ and other stakeholders’ engagement practices in the MNH improvement initiatives. Therefore, this review will focus on reviewing the different practices for stakeholders’ engagement, characteristics of stakeholder engagement (e.g., setting, diversity, type, and level of engagement), and outcomes of stakeholders’ engagement (e.g., improved service quality, delivery, and uptakes) in MNH research in the context of LMICs.

### Aim of the review

This study aims to systematically review the existing literature that includes service users and other stakeholders’ engagement in MNH research, particularly in LMICs. The synthesis of the included studies will answer three different key research questions.

RQ1: What ways are used for the engagements of service users and other stakeholders in maternal and newborn health research in LMICs?

RQ2: What are the characteristics of service users’ and other stakeholders’ engagement in maternal and newborn health services research in LMICs?

RQ3: What is known about the outcomes of involving service users and other stakeholders in maternal and newborn health research in LMICs?

## Methods/design

### Protocol design

The development of this systematic review protocol followed the Preferred Reporting Items for Systematic Reviews and Meta-Analysis Protocols (PRISMA-P) statement [37] (Additional file 1). The reporting of this systematic review will be guided by the Preferred Reporting Items for Systematic Reviews and Meta-Analysis (PRISMA) checklist [38]. The necessary modifications made in the protocol during the review process will be documented and published along with the systematic review results. This systematic review protocol is registered in PROSPERO, and the registration number is CRD42022314613.

### Search strategy and information source

The literature search will be conducted in PubMed/MEDLINE, PsycINFO, Scopus, Science Direct, and CINAHL, along with hand-searching references (citation chaining) for relevant scientific papers. The literature search in the different databases will be run using a search strategy developed by combining relevant keywords, controlled vocabulary, and Medical Subject Heading (MeSH) terms. The key concepts that will be used for the literature search are “service user”, ‘stakeholder’ ‘engagement’, “stakeholder engagement” “maternal and newborn health” “health service”, “service research” and “low and middle-income countries”. These concepts will further include the following sub-concepts/ MeSH terms (Table 1).

**Table 1:** Literature search concepts and sub-concepts/MeSH terms.

Search concepts	Sub-concepts/ MeSH terms
Service user	patient, mothers, family, consumer, client, relative, husband, “service user”, “pregnant mothers” “mother-in-law”, “caregiver”, “pregnant post postpartum women” “postnatal women” “lactating mother”, “new mother”, mother*, ‘neonate’, and ‘newborn’.
Stakeholder	public, provider, manager, health worker, doctor, nurse, midwives, policymaker, policy implementer, health assistant, volunteer, advocacy group, organization, NGO, INGO, leader, community, school, club, academia, researcher, committee.
Engagement	involvement, participation, participatory, “patient and public involvement”, PPI, collaborat*, engag*, partner*,“patient participation”, and user involvement”.
Types of research	community-based research, community-based participatory research, co-design, co-production, co-creation
Maternal and newborn health	“maternal health”, “newborn health”, “child health”, “antenatal service”, “antenatal checkup”, “institutional delivery”, “health facility delivery”, “postnatal checkup”, “postnatal services”, and “newborn health”, “neonatal health”, “prenatal care”, “perinatal care”, and “maternal mental health”.
Middle and low-income countries	“developing country”, “low income countries”, “low and lower middle income countries”, “low and lower-middle income countries”, “underdeveloped countries”, third world, list of LMIC individual countries as per the World Bank published report of 2021

The search strategy will be modified to each of the databases in agreement with investigators. Results from the databases will be exported to Endnote to avoid duplicates. Hand searches will be conducted via a bibliography search of full-text articles retrieved from the electronic database. Likewise, the reference list of reviewed papers will also be checked for potential papers that meet inclusion criteria.

### Selection criteria

Peer-reviewed articles published between January 1990 and March 2023 will be included. Only studies published in English will be considered. Only studies conducted in low and middle-income countries (according to the World Bank classification) will be included in the review. Research using any type of study design will be included in this review if it has evidence of service users or stakeholder engagement in any phase of the study to improve any aspects of maternal and newborn health services in LMICs.

### Condition or domain being studied

Literature including services users and stakeholders’ engagement in maternal and newborn health research, policy, or MNH literature, including program planning, implementation, or evaluation, will be considered for this study. Service user and stakeholder engagement in the review will refer to an active involvement or collaboration, or partnership among researchers, service users, and other stakeholders.

### Participants/population

Pregnant women, mothers of children aged under five years, husbands, family members, care-givers of mothers and newborns, and other professionals’ involvement in research for improving maternal and newborn health will be considered as participants or populations for the review.

### Intervention(s), exposure(s)

This review will include studies that have investigated the engagement of service users or other stakeholders or both, aiming to improve MNH services in different aspects. This includes quality improvements, service uptake and delivery improvements, availability, accessibility, affordability, decision-making process, awareness-raising, and promotion of maternal and newborn health and services in LMICs.

### Comparator(s)/Control

The comparator is not applicable for this review.

### Context

The review will include studies that considered the engagement of service users or stakeholders as a partner in any MNH service improvement process in the community and hospital settings in LMICs.

### Main outcome(s)

The main outcome of this study is to review the engagements of service users or stakeholders in the planning and development of MNH policies and programs, implementation, and evaluation of maternal and newborn services, or any kind of maternal and newborn research in LMICs. The study will also review the settings, types of service users, stakeholders, facilitators, procedure/methods, and intensity of users’ and stakeholders’ engagements in MNH research in LMICs.

### Additional outcome(s)

To assess the possible challenges, opportunities, barriers, and facilitators for involving users and stakeholders in MNH service research or improving health services in resource-poor settings.

### Data extraction (selection and coding)

This study will follow the Preferred Reporting Items for Systematic Reviews and Meta-Analysis (PRISMA) checklist [38]. The screening of the study title, abstract and search of the full-text article will be conducted by two reviewers. The quality, inclusion, and exclusion criteria for the study will be further independently assessed by reviewers. In case of disagreement, the final decision related to the exclusion or inclusion of the article in the review will be resolved by discussion among the reviewers. The articles selected to be included in the review will be extracted in the form of an electronic data table (Table 2). The title of the article, author list, publication year, study setting, study sites, methods used for user and stakeholder engagement in research, population characteristics, methods, and findings of the study will be extracted. The details of the data extraction template are provided in Table 2.

**Table 2:** Data extraction format.

Components	Types of information
Publication details	The title of the article, author list, country, and publication year
Study details	Objectives, study setting, study sites, study types, and purpose of user and stakeholder engagement
Types of user and stakeholder engagement	Ways of users and stakeholder engagement, level of engagement the study, types of stakeholders, and frequency of engagement
Outcomes of the engagements	Facilitators or barriers in terms of MNH improvements
Context of the engagements	Inner context (within the organization/institution) and outer context (outside the organization/institution)

### Risk of bias (quality) assessment

The methodology and quality of the paper will be assessed appropriately. The critical appraisal skills program (CASP) checklists [39] will be used to assess qualitative papers as a quality appraisal tool. Likewise, the Mixed Method Appraisal Tool (MMAT) checklist [40] will be used for the quality assessment of mixed-method or quantitative studies.

### Data synthesis

A narrative synthesis will be used to synthesise results from different design studies included in the review [41]. Data synthesis will be done in two steps, as suggested in the Cochrane Handbook of Systematic Reviews [42]. Firstly, the qualitative and quantitative evidence will be separately reviewed, and in the second step, all the evidence will be combined into an overall narrative synthesis. The Synthesis Without Meta-analysis (SWiM) reporting guidelines will be utilised to report the findings within this review [43]. This will support clearly presenting the rationale and process of how the studies are grouped, the use of synthesis methods to analyse the findings, the presentation of data and summary text, and the limitations of the review. In addition, we will use the standardised tables to house the main findings from each study included in the review. This will help to present a systematic summary of the studies, including tabulation and transformation of data into common practice, groupings and clusters, and textual descriptions. Similarly, the qualitative synthesis will explicitly describe the results, design, methods, and populations by extracting qualitative data using a data extraction form. Finally, the qualitative and quantitative data analysis will be combined to develop a narrative synthesis. The integrated synthesised results will be presented under the relevant themes identified during the data synthesis. Further, necessary analysis of the results can also be sub-grouped based on the users’ and stakeholders’ engagements in socially and ethnically disadvantaged communities to present the specific context and purpose of stakeholder engagements.

## Discussion

To our knowledge, this systematic review is the first of its kind that will present the synthesis of evidence from existing studies on service users and stakeholders’ engagement in MNH research, particularly in the context of LMICs. The evidence from this review may be an aid to the appeal of the WHO for stakeholder and community engagement in improving maternal, newborn, and child health [5].

This study’s results will help address the paucity of evidence in the related area by providing a comprehensive understanding of stakeholders’ engagements in MNH policy and program development, implementation and evaluation. It is argued that findings from well-conducted reviews are more likely to be utilised by decision-makers and researchers [44]. We will ensure methodological rigour at each stage of the review, and it will be guided by the Preferred Reporting Items for Systematic Reviews and Meta-Analysis checklist [38].

Thus, the persuasive results from this study will sensitise policymakers, planners, program implementers, and researchers to utilise the up-to-date evidence on service user and stakeholder engagements in MNH research. The study will also be helpful in finding knowledge gaps between the theoretical and empirical practice of service users and relevant stakeholder engagement in improving maternal and newborn health. The amalgamated evidence on potential facilitators and barriers in stakeholders’ engagements in resource-poor settings will enrich the researchers’ and practitioners’ understanding of appropriate and meaningful engagements of stakeholders in MNH policy and practice improvement. Therefore, we believe the findings from this study will certainly support improvement in designing people-centred MNH services in resource-poor settings.

## Supporting information

S1 Checklist PRISMA-P (Preferred Reporting Items for Systematic review and Meta-Analysis Protocols) 2015 checklist: Recommended items to address in a systematic review protocol*. (DOC)

## List of abbreviations

LMICs: Low- and middle-income countries
MNH: Maternal and newborn health
PPI: Patient and Public Involvement
SDGs: Sustainable Development Goals
WHO: World Health Organization
